# The Opening of the Medical Schools

**Published:** 1891-11

**Authors:** 


### THE OPENING OF THE MEDICAL SCHOOLS.

The medical schools of the country began the session of 1891–92, about one month ago, and work is now well under way. The opening of the two Atlanta schools was particularly auspicious and encouraging. It was thought and feared that the articles of agreement between the Atlanta Medical College, the Southern Medical College, and the Medical Department of the University of Georgia (see August number of the JOURNAL), whereby the tuition fee was to be raised to $100.00, and rigidly adhered to, would have the effect of greatly reducing the student attendance, and making many young men seek their instruction in other schools, which we might name, where they would be gladly received for the regular fee, if possible; for anything they could pay, if convenient; for nothing, if necessary. It is a source of pleasure and congratulation to the faculties of the Georgia schools that their fears have not been realized. The two colleges in this city never opened with better attendance, and were never so well prepared to do thorough and efficient work. The Atlanta Medical College and the Southern Medical College have each enrolled about as many men as were in attendance last session, and a few others will matriculate in each school. The new departure of the Georgia schools, above referred to, will greatly improve their clientele. We are pleased to note these indications of prosperity and progress. They are encouraging for the future medical education in the South.

It is still too easy a matter to obtain the medical degree. There are hundreds of men in college now, to be graduated next spring, and turned loose upon a too confiding community, who ought to be plowing a mule or carrying mortar. It is absurd for such "students" to attempt to learn any science, as they present themselves at our colleges, and most of all, the science of medicine. But they continue to receive their diplomas, the mere *possession of which*, in some States still—and Georgia must shamefully acknowledge the corn—qualifies the holder to practice his dangerous physic upon all those who unsuspectingly come within his reach.

In the matter of medical teaching two errors still exist in some of our colleges. The first is, that any man, with or without mental training and capacity, can pursue the study and practice of medicine. The second is, that any man, be he ever so bright, can qualify himself for practice by a two-term course of study, of five, or six, or seven months each. Happily, these illusions cannot last much longer. The spirit of reform and advance in our medical education is abroad now, and an enlightened profession and an enlightened public mind are very wisely demanding more of both colleges and students.

